# The Efficiency of Cooperative Learning in Physical Education on the Learning of Action Skills and Learning Motivation

**DOI:** 10.3389/fpsyg.2021.717528

**Published:** 2021-10-20

**Authors:** Cairu Yang, Rongli Chen, Xiaozhong Chen, Kuan-Han Lu

**Affiliations:** ^1^Department of Physical Education, Dongguan Polytechnic, Dongguan, China; ^2^Department of Computing Engineering, Dongguan Polytechnic, Dongguan, China; ^3^Department of Logistics Engineering, Dongguan Polytechnic, Dongguan, China; ^4^Department of Computer Science and Information Management, Soochow University, Taipei, China

**Keywords:** physical education, cooperative learning, learning motivation, action skills, effect

## Abstract

This paper proposes a cooperative learning method for use in physical education, involving two different grouping methods: S-type heterogeneous grouping and “free” grouping. Cooperative learning was found to enhance the effectiveness of basketball skills learning and learning motivation. A comparison was made of the differences between action skills grouping (the control group) and “free” grouping (the experimental group). The ARCS Motivation Scale and Basketball Action Skills Test were used to measure results, and SPSS statistical analysis software was used for relevant statistical processing (with α set to.05). The results showed that overall skills, dribbling and passing among the action skills groups and “free” groupings significantly improved, but results for shooting were not significant; motivation levels for the two grouping methods significantly improved overall, and no significant differences in learning motivation and learning effectiveness were found between the different grouping methods. It is clear that teachers should first establish a good relationship between and with students, and free grouping methods can be used to good effect. Teachers using cooperative learning should intervene in a timely manner and choose suitable grouping methods according to the teaching goals.

## Introduction

Physical education is based on physical activity and focuses on training physical fitness and improving health. In the past, physical education in schools was often regarded as marginalized or was not considered a suitable subject to study at higher education level, compared with other subjects. However, Spencer pointed out in the life preparation theory that the purpose of education is to prepare young people for a fulfilling and successful life in the future. Physical health and self-discipline are therefore highly important and are directly related to survival. The representative indirectly pointed out that the most valuable courses are those in the fields of health and physical education. In addition, many scholars have pointed out that in physical education, students can experience the fun of sports through sporting activities, and they can develop their sports skills along with personal and social skills. Sporting activities can help establish harmonious interpersonal relationships and enable individuals to develop appropriate ethical (sportsmanlike) and team behaviors, and they are an effective way to build self-confidence. Furthermore, exercise has the following benefits: it stimulates the brainstem and helps regulate neurotransmitters in the brain; it helps reduce anxiety and relieve stress; it improves strength; it can improve memory, learning ability and concentration; and it can promote feelings of well-being. The importance of physical education is therefore clear to see (Chen et al., 2019a; Bai et al., 2020).

Health has long been a topic of concern for people, and it has the most direct relationship with survival. In holistic terms, five elements of health and well-being are recognized: physical fitness, emotional fitness, social fitness, spiritual fitness and cultural fitness. Social fitness emphasizes active interaction with others and the ability to develop friendships. Ten basic abilities have been identified, associated with physical education, including respect, care and teamwork. The 12-year national education curriculum has developed the concept of “spontaneous,” “interactive,” and “shared good” in the nine years since its introduction, and the importance of this for children’s learning has been actively promoted in recent years.

The concept of teamwork and cooperation has always been valued, and the importance of cooperation has been mentioned many times in the major domains of education, and is reflected in fitness ability indicators. It can be seen that physical education can create an ideal context for cooperative learning. Acquisition of action skills in physical education is not something that happens automatically as children grow and mature, but it can be enhanced by external factors such as practice, guidance and encouragement. Cooperative learning provides these opportunities and is an effective teaching method that is advocated by experts and scholars. It can shape teamwork situations, and students can develop the ability to communicate, cooperate and coordinate with others in the process. Social skills can be enhanced at the same time, through mutual encouragement, teaching, explanation and other interactions between peers. Cooperative learning can stimulate individuals’ inner motivation, improve attitudes to learning, improve learning effectiveness and help young people achieve key learning goals. Many studies in the past have pointed out that cooperative learning can not only improve the effectiveness of learning but also make learning more enjoyable (Chen et al., 2015, 2021e, 2019b).

In addition, cooperative learning is also effective in promoting subject knowledge and problem-solving abilities. Assessing the effectiveness of learning has always been an important aspect of physical education (as with other subject areas), enabling teaching and learning outcomes to be evaluated and improvements and next steps to be explored further. Various factors will affect the effectiveness of learning. Studies of heterogeneous grouping have found that it helps to promote interaction between students, cultivate social skills, increase learning effectiveness and improve learning motivation. Cooperative learning almost equates with heterogeneous grouping. In physical education, the heterogeneous grouping method mostly involves skill-performance grouping, and most studies have pointed out that such a heterogeneous grouping method is beneficial to the learning of all group members. Better performing individuals can assist those with poorer skills so that the latter can obtain feedback on their performance and improve, and those with better skills can reorganize and improve their own performance by teaching other team members.

In this research, cooperative learning was applied to physical education classes in order to explore the impact of different grouping methods on learning effectiveness and learning motivation (Chen et al., 2020). Based on the research background and motivation, the aim of this study was to explore the impact of different cooperative learning grouping methods in relation to action skills learning and learning motivation. Quasi-experimental research methods were used so that the results could serve as a reference for physical education and future research.

### Research Questions

The following research questions were formulated:

(1) What differences can be seen (before and after testing) between action skills groups and “free” groupings in relation to the effectiveness of cooperative learning?
(2) What differences can be seen (before and after testing) between action skills groups and “free” groupings in relation to motivation in cooperative learning?
(3) What differences can be seen between action skills groups and “free” groupings in relation to performance of action skills in cooperative learning?
(4) What differences can be seen (pre- and post-test) between action skills groups and “free” groupings in relation to learning motivation in cooperative learning?

### Research Participants

(1) Research participants: Intentional sampling was used in this study. Two classes of students were selected as the research participants (59 participants in total). There were 29 students in class A (15 males and 14 females). Free grouping was adopted, and this was the experimental group in the research. Class B comprised 30 students (17 males and 13 females), grouped according to S-type heterogeneous action skills, and this was the control group.

(2) Research time: The implementation time for this research was from March 2019 to May 2020, a period of six weeks. There was a total of 12 lessons. One physical education lesson took place (45 min per class) every Monday and Thursday afternoon throughout the study period. The teaching strategy of cooperative learning was used to carry out physical education basketball teaching.

(3) Teaching content: In this study, a self-designed cooperative-learning teaching strategy was integrated into the basketball unit. The teaching content included basic basketball passing, dribbling and shooting.

(4) Research test restrictions: The ARCS Motivation Scale and the Basketball Action Skills Test were used in the pre- and post-tests of the study. Therefore, the pre-tests may have affected the post-tests.

### Interpretation of Terms

#### (1) Cooperative Learning

This is a structured and systematic teaching strategy to promote group learning. Group members participate in the learning together, and peers have a mutual relationship of success or failure and help one another to achieve the learning goal. For this research, two types of student group achievement differentiation (STAD) were used, and group game competition (TGT) to design teaching plans.

#### (2) Action Skills

In this research, the action skills consisted of dribbling, passing and shooting in basketball. Dribbling refers to the speed of the dribbling movement using the left and right hands; passing refers to the accuracy and speed of passing and receiving the ball bounced against the wall; shooting refers to the ability to shoot and pick up the ball (from the same distance) and shoot the ball into the basket.

#### (3) Different Grouping Methods

The different grouping methods in this study refer to free grouping within the limit set for the number of members and S-type heterogeneous grouping according to the performance of action skills. Participants were divided into five groups, with five to six people in each group. Once the groups had been set, they stayed as they were until the end of the study. The terms used for groups are defined separately as follows:

##### “Free” Grouping

Students could group themselves according to their own wishes, with at least five people in each group and a maximum of six people.

##### S-Type Heterogeneous Grouping of Action Skills

Action skills were defined as described above. The basketball skills test developed for the study was used as the test method. The pre-test scores obtained were ranked from high to low and were used as the basis for dividing the participants into five groups, each containing five to six people: 1∼5, 6∼10, 11∼15, 16∼20. The scores for the first group were 1, 10, 11, 20, 21, and 30. The scores for the second group were 2, 9, 12, 19, 22, and 29. From less to more, more to less, and so on.

##### Effectiveness of Action Skills Learning

Learning effectiveness is critical to teaching and learning outcomes. Learning effectiveness refers to the degree to which students achieve particular teaching goals. However, there may be a range of these, e.g., the main learning, auxiliary learning, cognition, motivation and skills. The action skills learning effect referred to in this research refers to the passing, dribbling and shooting scores obtained by students after the cooperative basketball lessons.

## Literature Review

### The Connotations of Cooperative Learning

Cooperative learning is a type of teaching. It means that two or more learners become a learning unit. Through the interaction of group members and the sharing of responsibilities, learners can achieve common learning goals. In this process, each learner must take responsibility for team members. This kind of teaching is learner-centered and can provide students with opportunities for active thinking and more interactive communication. Cooperative learning is a learning activity that establishes a common goal between group members, who then work together toward this, cooperate and support one another. Through the cooperation of peers, the effectiveness of individual learning is improved, and group goals can be achieved. For learning to be fully cooperative, it needs to have the following three key elements: promotion of positive interdependence; personal performance responsibility; face-to-face interaction. Cooperative learning is a structured and systematic teaching strategy, which is less subject to the restrictions of subjects and grades. Teachers can help meet the needs of students of different genders, abilities, socioeconomic backgrounds, races, etc.

After getting into groups, the whole group establishes a common goal. All group members are responsible for themselves and for the others. They encourage and assist one another in order to achieve the learning goals. Teachers arrange suitable cooperative learning situations and group students in a heterogeneous manner, providing guidance to students to help them cooperate, learn from one another, share resources and achieve learning goals together, which not only contributes to learning achievement but also to motivation levels. Cooperative learning is a systematic and structured teaching strategy, which supports learning outcomes and also students’ communication and social skills (Chettaoui et al., 2020; Chen et al., 2021d, e).

### Cooperative Teaching Methods

Teachers can choose appropriate teaching methods to apply in the classroom according to teaching goals, student characteristics and the characteristics of the subject being taught. Cooperative learning methods are divided into three categories, according to the teaching situation. The first type is communication, which focuses on sharing (and discussion) of ideas among group members; the second type is that of proficiency, which focuses on the content of the course; and the third type is inquiry, which focuses on guiding groups as they explore set tasks and solve problems. Below, five other methods commonly used in physical education will be introduced, along with the student group achievement differentiation (STAD) method used in this research.

#### Students’ Team Achievement Differentiation Method

The student group achievement differentiation method is the most straightforward and easy-to-implement cooperative learning method. This implementation and evaluation method is similar to traditional teaching methods, but it also has other special benefits, such as group rewards, individual responsibilities and equalization. The test method ascertains the progress scores of each group member and the entire group, meaning that the effort and achievement of all students can be recognized and celebrated. Progress scores are used to confirm the extent to which teaching goals are achieved. The STAD process may involve whole-class teaching, group study, quizzes and calculation of personal progress scores. For the purposes of this study, participants’ individual scores (before cooperative learning) were calculated for each group member, and the detailed score comparison is shown in Table 1.

**TABLE 1:** Personal progress score conversion table.

Quiz-Basic score	Score
Improved by more than 5 points, outstanding performance	15
Progress 0-4 points	10
Step back 1–5 min	5
Regress by more than 11 points	0

#### Research Relating to Cooperative Learning

Cooperative learning began to develop after establishment of the Cooperative Learning Centre. Many experts and scholars have successively proposed cooperative learning-related teaching strategies and methods, and related research has also continued to develop. The research objects range from kindergartens to colleges and universities. In the field of sports, colleges and universities are the main users. A meta-analysis of relevant literature on cooperative learning showed that 80% of the results indicate that cooperative learning can have a positive impact on learning effectiveness; 13% of the results showed that there is no difference between cooperative learning and general teaching methods; 12% of the results showed use of the one-class teaching method, which has better learning results than cooperative learning. From the above meta-analysis, it was found that not all cooperative learning has positive effects. This research explored the relationship between cooperative learning and physical education. From previous literature, it was found that cooperative learning can be used in different projects and different stages of learning, and it can be combined with other teaching methods so that students’ cognition, skills and motivation can be enhanced (Chou et al., 2015; Chiang and Yang, 2017; D’Aniello et al., 2020; Ding et al., 2020).

Not all results support the positive impact of cooperative learning in physical education classes, but few studies have found there to be no positive impact. Most of the results show that cooperative learning is a highly feasible teaching method and can be used not only in team sports such as baseball, football and volleyball but also in activities involving a small number of people or individual sports such as tennis, badminton, billiards and gymnastics. Outcomes are not dependent on the learning stage, and cooperative learning is suitable for all ages of students, including college students.

In the past, related research variables applied to physical education classes included learning effectiveness, learning motivation, interactive behavior, critical thinking, physical activity, etc. Among these, learning effectiveness has received the most attention. Not only has the learning effectiveness of action skills been found to improve but also interpersonal, communication and social skills. Furthermore, personal motivation is stimulated during interaction (Dyson, 2001, 2002).

Studies have shown that cooperative learning can improve learning effectiveness, physical activity and learning motivation more than traditional independent learning methods. Previous research into application of cooperative learning in physical education classes has yielded promising results, showing that cooperative learning is more efficient than learning in isolation or competitively. Cooperative learning is a well-established teaching method, and it is a common strategy in the field of research and teaching. Since most studies show the positive impact of cooperative learning, how to bring the greatest positive impact using this learning strategy is worthy of in-depth discussion (Dyson et al., 2004; Chen et al., 2021a).

### ARCS Motivation Scale

ARCS learning motivation theory is based on comprehensive integration of different forms of learning motivation and related theories in the United States, such as cognitive school attribution theory, behavior school reinforcement theory and other theories proposed by the motivation model. This theory is based on the premise that learners’ internal psychological factors, teachers’ teaching designs and learning effectiveness are closely related. These are important factors affecting the effectiveness of learning. It is believed that traditional teaching designs have, in the past, ignored learners’ motivation for learning. If learners are not interested or are unable to focus on learning, the effect of learning will be greatly reduced. The ARCS motivation model provides teachers with a better understanding of students’ motivational needs, so that they can design courses based on learners’ needs in order to stimulate learning motivation and enhance learning effectiveness. ARCS constitutes a relatively complete set of motivational factors. It is not restricted by age and is applicable to all learning stages. Therefore, the ARCS Motivation Scale is often used to investigate student learning motivation. ARCS stands for “Attention,” “Relevance,” “Confidence,” and “Satisfaction,” which are key to learning that stimulates motivational levels and attracts the attention of learners (Fu et al., 2018).

Students’ interest is linked to the perceived “relevance” for them personally and to feelings of “self-confidence” in terms of students’ perceived ability to achieve their goals. Finally, it is important for students to feel a sense of “satisfaction” from the learning process. ARCS emphasizes that in order to arouse students’ learning motivation, the above four elements must be provided for in order for teaching to be effective.

### Grouping Method for Physical Education Classes

An important step before implementation of teaching in cooperative learning is to group students. It is important to group students appropriately so that they will not resist psychologically and to ensure that there is a good interactive relationship between group members, with all group members willing to work together for the group. The goal is to work hard to achieve the desired learning outcomes, so how to organize groups is a major issue in cooperative learning. For middle-school children, the distribution method normally used is to divide up classes, so groups will be uneven, with large differences. In this situation, cooperative learning usually involves heterogeneous grouping, so that students with different characteristics are allocated to each group. This can serve to even out individual shortcomings, so that each group will have its own merits, while also reducing the adverse effects caused by individual differences. As “free” grouping is very straightforward, there is no need to do any preparatory work, and students can stay with their friends. Therefore, the method of letting students select their own group members is frequently used on campus. In cooperative learning, the members of the group will be affected by the way the group is formed. A good grouping method can make the team work harder toward the common goal and significantly improve learning (Gillies, 2004; Goodyear et al., 2014).

Before considering the subject of heterogeneity grouping, another term should be briefly explained, namely homogeneity. This means that two or more individuals have certain attributes or traits that are similar. These attributes or traits may refer to the level of skills, motivation, education or social and economic background. Heterogeneity is the opposite of the above and refers to differences in certain attributes or traits between two or more individuals. According to the definition of heterogeneous grouping, it is believed that cooperative learning with heterogeneous grouping can bring together students with diverse characteristics (such as background, abilities, experience and interests), so that they can learn from others with different attributes during the learning process. Being exposed to different ideas and perspectives will stimulate cognitive imbalances and challenge learners’ knowledge structures, thereby generating new knowledge.

In the past, there have been many studies comparing cooperative learning with other teaching methods, and it has generally been found to be a reliable method. Most of the above-mentioned studies point out that heterogeneous grouping can improve student performance in cooperative learning, and such studies have been based on the hypothesis that heterogeneous grouping yields significantly better results. There are many types of heterogeneous grouping methods. When heterogeneous grouping is applied to physical education, groups can be based on the following: skills or technical ability, gender, learning style, learning motivation, height and weight, and sporting expertise. Physical education is a subject that emphasizes action skills. Therefore, heterogeneous grouping will be based on performance of action skills (although some studies have focused on implementation of grouping based on other perspectives) (Huang et al., 2012; Hernandez et al., 2020).

### Study of Learning Effectiveness in Physical Education

Learning outcomes can be evaluated following teaching, so that students can better understand their own learning, and teachers can review their practice and endeavor to improve students’ test results. The three educational goals evaluated in this study (in relation to physical education classes) were cognition, motivation and skills. In physical education classes, cognitive measurements can involve oral reports, observations or paper and pencil tests. Test content usually relates to the rules and strategies of each sport, sports development history and general knowledge about physical fitness; motivation is scored according to sports participation, effort, learning attitude, class attendance, etc.; and skill level is based on the skills of each sport. Evaluation methods used in physical education classes particularly focus on development of sports skills, and the focus of this study was skills learning in basketball (Hung, 2004; Huang et al., 2017).

#### The New Direction of Physical Education Assessment

In the past, assessments were based on the three categories of cognition, motivation and skills. In the future, in order to improve the consistency of evaluation standards in the field of fitness, a new type of teaching target (that of “behavior”) will be classified in evaluations. In addition, in the national middle-school learning evaluation standards currently being piloted, the following sub-themes (for physical education) are included: sports knowledge, skill principles, learning attitude, sports appreciation, skill performance, tactical application, sports planning and sports practice, etc. Physical education evaluations should serve to help students improve their ability to perform key skills. Other aspects of teaching should be adjusted according to actual teaching considerations, and (taking account of the differences in students’ abilities) the “process of hard work” in students’ learning should be understood in a diversified manner. The grades traditionally used (such as excellent, A, B, C, D, etc.) were changed to five A to E grades, based on student performance: A indicated “excellent”; B was “good”; C was “fair”; D was “inadequate”; and E indicated “below the required level”.

#### Evaluation of Basketball Learning Effectiveness

The relationship between evaluation and teaching objectives is inseparable. An evaluation design must be based on teaching objectives and the principle of segmented ability indicators. There are many ways to evaluate learning outcomes, depending on the purpose and target, with different timings and different use cases. Basketball is one of the school’s main teaching programs and is a popular sport. Although general physical education classes can use objective and subjective evaluation methods, secondary evaluations are more suitable for research and should be neutral and objective to avoid being affected by subjective factors. Therefore, the subjective evaluation method was not used in this study, and we adopted a single-objective evaluation method to evaluate teaching content (basketball skills of dribbling, passing and shooting). Not only is this method of assessment suitable for the teaching content, but the applicable objects also conform to the teaching objects of this time. In addition, if the cooperative learning strategy is used to teach basketball, whether the target is elementary school, junior high school or college students, or students with low sporting achievement in junior high schools, it can effectively improve basketball skills performance and acquisition of essential basketball knowledge. In terms of performance and students’ understanding of basketball strategy, it can be seen that application of cooperative learning in this context is feasible and can have a positive impact (Huxham and Land, 2000; Ibarra et al., 2019; Chen et al., 2021b).

### Research Hypothesis

From the literature review, it was found that cooperative learning can have a positive impact on learning effectiveness and learning motivation, and free grouping and action skills grouping have been used in cooperative learning. Both grouping methods have advantages and disadvantages. Although differences between the two groups have been compared in the past, it could not find any comparison of the two grouping methods in physical education reported in the literature. No studies have yet been carried out on free grouping in physical education. Therefore, the following research hypotheses were formulated for the research purposes and experiments:

Hypothesis 1: In cooperative learning, skills-based grouping and free grouping yield significantly better post-test results relating to the effectiveness of action skills learning.

Hypothesis 2: In cooperative learning, skills-based grouping and free grouping yield significantly better post-test results relating to motivation for learning.

Hypothesis 3: In cooperative learning, there is no significant difference between the effectiveness of action skills learning in skills-based groups or free grouping.

Hypothesis 4: In cooperative learning, there is no significant difference between learning motivation in skills-based groups and free grouping.

#### Difference Between of Free Grouping and Other Grouping Methods

Free grouping can achieve teaching goals because of the friendship factor. In this study, middle-school students were divided into three groups: mixed-ability and homogeneous grouping; S-type heterogeneous grouping; and free grouping. It was found that free grouping had obvious learning effects in terms of cognition, motivation and skills. Post-test results for learning motivation were significantly better than those for the research hypothesis 1 and 2 of the pre-test. With free grouping, students with medium and low abilities improved their cognition, motivation and skills. With these three grouping methods, high-ability students can achieve cognitive, affective and technical learning(Huang et al., 2004; Chen et al., 2021c).

#### No Difference Between Free Grouping and Other Grouping Methods

The study of the impact of the three grouping methods (“heterogeneous grouping,” “homogeneous grouping,” and “free grouping”) in the natural sciences in school in terms of learning effectiveness found no significant difference in overall academic performance. The students were divided into heterogeneous groups and self-chosen groups. The results of the study indicated that there was no difference in learning effectiveness between heterogeneous grouping and self-grouping. It was found that the three groups (“heterogeneous grouping,” “homogeneous grouping,” and “free grouping”) could all improve the academic achievement of students. Based on the above research results, research hypothesis 3 was formulated, i.e., that there would be no significant difference between the two groups in relation to action skills learning. In the above research on free grouping, it was found that the results are not yet stable (although free grouping has been evaluated in many fields) (Huang et al., 2006, 2015, 2019b).

In addition, the way the group operates will have an impact on students’ cognition and motivation and the effectiveness of skills learning. Relevant studies in the literature have found free grouping to be effective. In addition to improving cognitive skills, it also has other beneficial effects (e.g., on attitude and cohesion), and it is particularly effective for low-achieving students. Based on the above research results regarding attitudes to learning, this paper hypothesized that there would be no significant differences between the two grouping methods in terms of learning motivation (hypothesis 4) (Huang et al., 2019a).

## Research Methods

### Research Structure and Process

This study mainly explored the influence of different grouping methods on the effectiveness of cooperative learning and motivation in physical education. The independent variable was grouping. The experimental group used free grouping, and the control group was based on skill levels. In order to avoid affecting the results, the control variables were teaching method, teaching time, course content, teacher characteristics and teaching environment. The dependent variable mainly explored differences between the experimental group and the control group in terms of the effectiveness of action skills learning and motivation (Kang, 2019; Laurens and Valdés, 2020).

#### Research Structure

**FIGURE 1:**
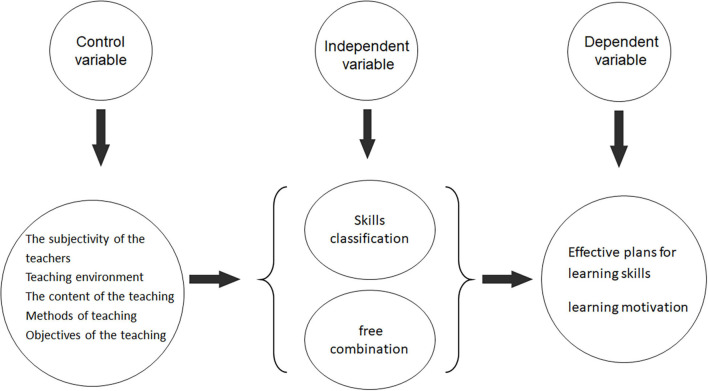
Research architecture diagram.

The research framework was based on the research background, research purpose and research questions, and it was drawn up based on the results of the literature review. The control variables in this research framework were teaching methods, teaching time, course content, teacher characteristics and teaching environment; the independent variables were grouping methods (free grouping and skills-based grouping); the dependent variables were the effectiveness of action skills learning and learning motivation. The research architecture diagram is shown in Figure 1 below:

#### Research Design

This study used ARCS motivation grouping and skills grouping as independent variables, and ARCS learning motivation and action skills as the experimental teaching of dependent variables, in an attempt to compare the influence of grouping on learning motivation and action skills learning. In the research design, T, ARCS Motivation Scale; Action Skill Test X1, free grouping; X2, skill grouping, as shown in Table 2.

**TABLE 2:** Research design.

Group	Pre-test	Control variables	Post-test
E (experimental group)	T	X1	T
C (Control group)	T	X2	T
			

##### (1) Students Participating

The students participating in the study had the same physical education teacher in the first grade, and the research teacher took over in the second grade. The pre-test results were close for both types of grouping. Before the formal start of the experiment, the two groups of students took the basketball skills performance test and the ARCS Motivation Scale pre-test. Once the two groups of teaching experiments were complete, the students immediately retook the basketball skills performance test and the ARCS Motivation Scale post-test. The statistical table of class size is shown in Table 3. Our analysis of the relationship between gender and group distribution for the 59 study participants showed that the distribution ratio of male to female students was 54.5%:45.5%. (Respondents who completed the questionnaire in Appendix).

**TABLE 3:** Class size statistics.

Group	Grouping	Male	Female	Total
Experimental group	Free grouping	15	14	29
Control group	Skill performance	17	13	30

The chi-square test was used to analyze the results for gender and group and found that *x*^2^ = 0.16, *p* = 0.69 > 0.05, which did not reach the significant level, indicating that there were no significant differences. The differences are shown in Table 4.

**TABLE 4:** Gender test summary table.

	Numerical	Degree of freedom	Significance
Pearson Chi-square	0.16^a^	1	0.71
Continuity correction	0.02	1	0.92
Number of valid observations	59		

*^*a*^0 cells (.0%) have expected count less than 5.*

##### (2) Sample Homogeneity Test

This test was to understand whether there were significant differences in the test scores for the “Basketball Skills Performance Test” and “ARCS Motivation Scale” between the action skills groups and free groupings before the experiment, which might have caused errors in the research results. An independent sample *t*-test was conducted based on the pre-test scores for the two groups, and the data were analyzed. If a significant difference was found, a covariate analysis was carried out to establish the equality between the two groups and the post-test.

With regard to the action skills grouping and free grouping ARCS motivation pre-test homogeneity test, the test scores for the experimental group and the control group are shown in Table 5. It can be seen that the results of the homogeneity test for the control group and the experimental group in terms of learning motivation (before testing) were not significant for overall learning motivation, self-relevance and satisfaction (*F* = 2.80, 1.68, 0.48, *p* > 0.05), but the results were significant for attention and self-confidence (*F* = 1.68, 1.06, *p* < 0.05). The relevant parameters of the mean and standard deviation of learning motivation were 2.93 ± 0.31 and 3.08 ± 0.36. The components were as follows: intent: 2.98 ± 0.46 and 3.36 ± 0.43; perceived relevance: 3.22 ± 0.49 and 3.35 ± 0.53; self-confidence: 2.96 ± 0.60 and 3.33 ± 0.61; satisfaction: 3.31 ± 0.56 and 3.43 ± 0.54.

**TABLE 5:** Homogeneity test of action skill grouping and free grouping ARCS learning motivation pre-test.

Variable	Group	Mean	Standard deviation	Degree of freedom	t	*F*-test	Significance
Learning motivation	Experimental group	2.92	0.32	54	–1.6	2.8	0.10
	Control group	3.06	0.35				
Attention	Experimental group	2.95	0.45	54	–3.1	1.6	0.01
	Control group	3.33	0.44				
Self-associated	Experimental group	3.24	0.47	54	–0.88	1.0	0.35
	Control group	3.31	0.55				
Self-confidence	Experimental group	2.90	0.58	54	–2.2	0.73	0.04
	Control group	3.35	0.57				
Satisfaction	Experimental group	3.30	0.60	54	–1.9	0.45	0.03
	Control group	3.45	0.55				

#### Homogeneity Test for Basketball Skills (Pre-test) in the Control Group and the Experimental Group

The pre-test scores for the control group and the experimental group in terms of basketball skills performance are shown in Table 6. It can be seen from Tables 2–4 that there were no significant differences between the control group and the experimental group in terms of performance of basketball skills and the homogeneity test results for overall basketball skills, shooting and passing tests (*F* = 0.14, 7.18, 3.33, *p* > 0.05), but there was a significant difference in dribbling (*F* = 2.3, *p* < 0.05). The average basketball skills of the experimental group and the control group were 56.43 ± 12.13 and 50.93 ± 11.19, respectively; the average number of shots was 11.25 ± 3.33 and 12.46 ± 5.56; average passing was 28.58 ± 9.14 and 25.20 ± 7.18; and average dribbling was 16.18 ± 2.22 and 13.20 ± 3.23.

**TABLE 6:** Homogeneity test of pre-test of action skill grouping and free grouping in basketball skill performance.

Variable	Group	Mean	Standard deviation	Degree of freedom	*t*	*F*-test	Significance
	Experimental group	56.39	12.14				
Overall skills				54	1.67	0.16	0.08
	Control group	50.88	11.18				
Pitch	Experimental group	11.28	3.33	54	–0.86	6.09	0.33
	Control group	12.55	5.5				
Pass	Experimental group	28.69	9.04	54	1.57	3.23	0.10
	Control group	25.22	7.16				
Dribble	Experimental group	16.30	2.16	54	3.89	2.19	0.00
	Control group	13.13	3.21				

#### Homogeneity Test Results

According to the test results, there were no significant differences between the action skills group and the free groupings in terms of overall learning motivation, self-relevance and satisfaction; and overall basketball skills, passing and shooting were also not significantly different. This means that the two groups of subjects had homogeneity before commencing the cooperative learning research, and the independent sample *t*-test could be used directly. However, attention, self-confidence and basketball skills performance in learning motivation had significant differences in the pre-test, so single-factor covariate analysis was used for the post-test results to adjust for the differences (Lin et al., 2020).

### Research Tools

The tools used in this research included five items: a stopwatch for timing, a technical ability test, the ARCS Motivation Scale, a checklist relating to cooperative group learning and a teacher checklist. These tools are explained in the experimental equipment table below.

#### Basketball Skills Performance

##### (1) Movement Teaching

The main basketball skills focused on in the teaching project were basic ball sense, dribbling, passing and shooting.

##### (2) Basketball Skills Test Method

**FIGURE 2:**
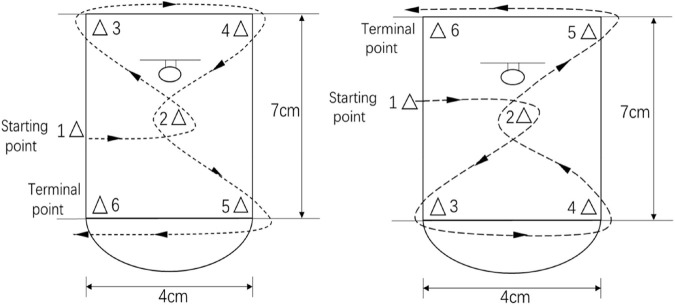
Dribbling test diagram. In the above illustration, on the left is the left-hand ball; the context is a specified basketball court; the specified time and the ball route are shown by the dotted line.

Two approaches can be adopted for assessment of students’ action skills learning: objective skill assessment and subjective skill assessment. Objective skill assessment involves measurement of distance or time with a measuring tape, stopwatch or by counting. Subjective skill assessment involves considering the pros and cons of postural performance, such as pitching power, posture and coordination. In this study, we used the basketball skills test developed for the study (see Figure 2).

*Test content* The pre-test and post-test units for action skills learning effectiveness in this research entailed three tests of basketball skills (dribbling, passing and shooting).

*Test subjects* The test tool for this research is suitable for use with 10- to 22-year-old subjects and is not restricted by gender. It is appropriate for use with the participants in this study.

*Difficulty of distinguishing* The average difficulty index of the test tools in this research was.65∼0.95.

*Internal consistency* The internal consistency α coefficient of the test tool in this study was between 0.84 and 0.97.

*Scorer reliability* Scorer reliability needs to be above 0.80 to be reliable. In this study, the internal scorer reliability was 0.94 and 0.91, and the inter-rater reliability was 0.90.

#### ARCS Motivation Scale

This research used the ARCS Motivation Scale. The scale is divided into two parts and contains 17 questions, which are explained as follows:

(1) Basic information relating to students: such as gender, exercise habits, previous semester’s sports score, physical fitness level and whether students have participated in school sports teams in the past (five questions in total).
(2) Learning motivation in physical education: This research used the ARCS Motivation Scale, which was a revised version of the designed learning motivation scale. The internal consensus reliability for “Attention” was 0.84; “Relevance” was 0.80; “Confidence” was 0.79; “Satisfaction” was 0.89; and the overall internal consensus was 0.96. This tool uses a Likert formula four-point scale (four-point Likert scale), as follows: 1 means “strongly disagree”; 2 means “disagree”; 3 means “agree”; and 4 means “strongly agree.” The four factors in ARCS are concentration, relevance, self-confidence and satisfaction.
(3) Attention: This relates to students’ level of curiosity or interest, and the aim of teaching is to ensure that this is maintained.
(4) Relevance: This relates to students’ perception that the content of the course or teaching activities relate to their own life, needs, familiar things or past experience, or that the learning may come in handy in the future.
(5) Confidence: This relates to students’ mental state in terms of whether or not they feel they can achieve the learning goals, which will, in turn, affect the actual degree of effort expended by students and their performance level. It is helpful if students believe that there is a link between success and effort.
(6) Satisfaction: This relates to positive inner feelings and also external rewards that students receive in the learning process; this kind of satisfaction is an important factor that helps sustain motivation.

Question numbers: attention: 1, 2, 3; relevance: 4, 5, 6; confidence: 7, 8, 9; satisfaction: 10, 11, 12.

### Teaching Design

The participants in this study were divided into two groups: the experimental group and the control group (based on two classes). The experimental group adopted free grouping, and the method used for the control group was skills-based grouping. The teaching experiment lasted for eight weeks. In the first two sessions of the formal class, the pre-test was conducted, and an explanation of the research was given. The last two sessions involved the post-test. Every Monday and Thursday afternoon throughout the study, the students participated in a session of physical education (45 min per class), involving cooperative learning, focusing on basketball skills. There were 16 lessons in total, including the two pre-tests and two post-tests.

#### Preparation Before Teaching

(1) Pre-test and grouping: Free grouping: Before the experiment began, students in the experimental group were allowed to choose their groups, with a maximum of five to six people per group. Groups within the control sample were formed based on the students’ pre-test scores for basketball skills. The highest and lowest scores for each ability were grouped in the same group, and the second highest and second lowest scores were grouped in the same group. By analogy, the class was divided into five groups. Immediately after the grouping, each group was asked to come to an agreement within each group. Each student was to express his or her opinions and work toward the goal, together with the others in the group.

(2) Roles and task assignment: According to the content of the learning task, the members of each group were to take turns at playing each role. Except for the captain’s role, the other roles could be played by two or more people at the same time.

(3) Establishing a tacit understanding: Before the teaching experiment, students underwent cooperative learning and interactive skills training, such as teamwork, communication skills, leadership skills, maintenance of an atmosphere of mutual trust and conflict resolution.

#### Teaching Implementation Stage

The teaching implementation phase of this research was divided into three parts: preparatory activities, development activities and comprehensive activities. At the same time, based on the steps of cooperative learning, whole-class teaching, group learning, individual performance and team history, the three main skills were shooting, dribbling and passing in basketball. The teaching module comprised the following: one class to establish group relationships; three classes for the shooting unit; and four classes for each of the dribbling and passing units. There were 12 lessons in total. The main teaching method used in the cooperative learning was student group achievement differentiation (STAD), supplemented by the group game competition (TGT) method for design of teaching plans.

#### After the Teaching Stage

Following delivery of the teaching module, a review was completed to reflect on the following: whether the teaching methods for the two groups had been the same; whether the teaching objectives had been achieved; whether the teaching plan had been carried out according to the teaching plan; whether the teaching plan had been properly designed; and whether the elements of cooperative learning had been provided for.

### Data Processing

The data processing method used in this research involved the SPSS statistical software package, to analyze the action skills test scores and learning motivation levels for the different groups after the students had completed the cooperative learning.

(1) Dependent sample *t*-test: This tested the difference between free grouping and action skills grouping after cooperative learning (comparison of “before” and “after” test results).
(2) Independent sample *t*-test: This tested the difference between free grouping and action skills grouping after cooperative learning (comparison of “before” and “after” test results for motivation).
(3) Covariate analysis: For the free grouping and action skills grouping, respectively, in the performance of the previous test, the difference between the two did not reach a significant level. The independent sample *t*-test was then used to analyze the difference. The difference between the two pre-test scores was found to reach a significant level. The previous test scores had common variables, and a co-variable analysis was performed to adjust the differences.
(4) In the above statistical analysis, the significance level of all the differences tested was set as α = 0.05.

## Results and Discussion

The data collected from the experiments were used for statistical analysis and discussion.

### Difference Between Action Skills Learning in the Control Group and the Experimental Group Before and After Cooperative Learning

Grouping in the control sample was skills-based. The differences between the pre-test and post-test scores were analyzed by an independent sample *t*-test, as shown in Table 7. The results indicated that overall basketball skills, passing and dribbling skills were significantly different (*t* = −3.60, −3.46, 4.70, *p* < 0.05), but a significant difference was not observed in the case of shooting (*t* = −1.21, *p* > 0.05). The post-test results for overall skills, shooting, passing and dribbling were all higher than the pre-test scores (*M* = 50.77 < 63.90, 12.50 < 14.45, 25.12 < 32.14, 13.18 < 17.12).

**TABLE 7:** *t*-test analysis of the basketball skill performance pre-test and post-test repeated measures of the control group.

Variable	Test	Number	Mean	Standard deviation	Degree of freedom	*t*-Test	Significance
Overall skills	Pre-test	30	50.77	11.09	54	–3.60	0.00*
	Post-test	30	63.90	15.24			
Shooting	Pre-test	30	12.50	5.50	54	–1.21	0.23
	Post-test	30	14.45	6.15			
Pass	Pre-test	30	25.12	7.14	54	–3.46	0.00*
	Post-test	30	32.14	8.11			
Dribble	Pre-test	30	13.18	3.07	54	4.70	0.00*
	Post-test	30	17.12	2.44			

*p < 0.05.

#### Discussion of Differences Between the Control Group Before and After Cooperative Learning

Cooperative learning using skills-based grouping can effectively improve skill performance. In the past, cooperative learning using heterogeneous grouping in various sports has been shown to improve the effectiveness of action skills learning. The results of this study were found to support research hypothesis 2. It may be inferred that this is to do with the heterogeneous grouping of action skills. Highly skilled performers in each group can rectify incorrect actions in time and give feedback, and the interdependence of goals and tasks in the elements of cooperative learning are such that everyone must contribute and take responsibility for achieving the goals of the group. In heterogeneous grouping, each group will have some students with strong action skills, who can act as a model, so that other students with weaker action skills can imitate them and learn from them, adjusting their actions accordingly, thereby increasing the effectiveness of action skills learning.

However, of the basketball skills studied, the improvement in shooting was not found to be significant. This may have been due to the high level of uncertainty associated with shooting. Even the most powerful players cannot achieve a 100% success rate for shooting, and shooting takes a long time. Therefore, although the improvement was not significant in statistical terms, this may be due to the limitations of the teaching environment for the three skills involved. Passing and dribbling just require an open space, but shooting is restricted by the venue, and teaching activities can only be carried out in a venue with a basketball hoop. Under this limitation, the number of shooting courses was reduced to one lesson. In addition, in the research and in our observations, it was found that the heterogeneous grouping was due to the large gap between the strengths and weaknesses of group members in the same group, so that students with strong action skills could not play to their fullest and lacked the feeling of competing at their highest level. In the post-test, the participants wanted to complete the test as soon as possible, possibly shooting without aiming carefully enough (Lu et al., 2005; Liu et al., 2014; Nikou and Economides, 2018).

#### Differences in the Experimental Group Before and After Cooperative Learning

The experimental group used the free grouping method. The results of the independent sample *t*-test are shown in Table 8. Our findings indicated that overall basketball skills, passing and dribbling skills were significantly different (*t* = −3.40, −3.18, −4.87, *p* < 0.05), but there was no significant difference in shooting (*t* = −1.03, *p* > 0.05). Post-test scores for overall skills, shooting, passing and dribbling were all higher than those of the pre-test (*M* = 56.44 < 67.47, 11.33 < 12.39, 28.72 < 36.20, 16.23 < 18.88).

**TABLE 8:** The basketball skill performance pre-test and post-test repeated measurement *t-*test analysis of the experimental group.

Variable	Test	Number	Mean	Standard deviation	Degree of freedom	*t*-Test	Significance
Overall skills	Pre-test	29	56.44	12.14	52	−3.40	0.00*
	Post-test	29	67.47	11.09			
Shooting	Pre-test	29	11.33	3.33	52	−1.03	0.31
	Post-test	29	12.39	3.15			
Pass	Pre-test	29	28.72	9.10	52	−3.18	0.00*
	Post-test	29	36.20	8.11			
Dribble	Pre-test	29	16.23	2.22	52	−4.87	0.00*
	Post-test	29	18.88	1.45			

*p < 0.05.

#### Discussion of Differences Between Pre-test and Post-test Results for the Experimental Group

The experimental group was based on free grouping, and this grouping method could significantly improve students’ overall basketball skills, passing and dribbling. The results of this research supported research hypothesis 1. It was found that free grouping could improve the effectiveness of action skills learning. The researchers inferred that with self-chosen groups, many of the students could stay with their friends, whoever they were, and they were more willing to actively assist and help. In a good relationship, students are more patient and supportive. They are also more willing to learn, and the friendship factor in free grouping can make it easier for students to achieve the teaching goals. In addition, self-grouping can help students obtain benefits other than those explicitly intended by the teacher. Such behaviors can further improve the effectiveness of action skills learning. In the research results, although the shooting scores improved, they did not reach a significant level. The researchers deduced that factors which might have affected these results included the difficulty of acquiring and consolidating such skills and the relatively small number of lessons spent on developing this skill (Ratnaningsih et al., 2020).

Another factor may be the order of learning. Shooting was taught before the other movement skills, but it could take about one and a half months to master. If students had not reached the automatic stage by the time of the post-test and had not been practicing this skill for very long, the retention effect after learning may have been poor. Another reason may be that shooting was the first item to be introduced after the free grouping. At this point in time, the students might not yet have become fully engaged with their studies. In addition, the group members, who were mostly all students of their own choosing, would have had plenty (possibly too much) to talk about, with common interests, etc., so at the beginning of the practice, there may have been more chatting and off-task behavior, reducing the practice time.

### Differences in Motivation Levels Between the Control Group and Experimental Group Before and After Cooperative Learning

To explore the pre-test and post-test differences in learning motivation between the control group and the experimental group (before and after cooperative learning), a statistical analysis of the dependent sample *t*-test was carried out.

#### Differences in the Control Group Before and After Cooperative Learning

The control sample, grouped according to skill levels, was tested by an independent sample *t*-test before and after cooperative learning, as in Table 9. It was found that overall learning motivation was significantly different (*t* = −2.48, *p* < 0.05), but attention, perceived relevance, self-confidence and satisfaction were not significant (*t* = 0.54, −0.57, −0.52, 0.39, *p* > 0.05). Attention and satisfaction scores were higher than in the pre-test (*M* = 3.33 > 3.29, 3.46 > 3.37); the post-test scores for perceived relevance and self-confidence were higher than those of the pre-test (*M* = 3.38 < 3.46, 3.30 < 3.43).

**TABLE 9:** Control group ARCS learning motivation scale pre-test and post-test repeated measures *t*-test analysis.

Variable	Test	Number	Mean	Standard deviation	Degree of freedom	*t*-Test	Significance
Learning motivation	Pre-test	30	3.08	0.36	54	–2.48	0.02*
	Post-test	30	3.38	0.52			
Attention	Pre-test	30	3.33	0.43	54	0.54	0.58
	Post-test	30	3.29	0.53			
perceived relevance	Pre-test	30	3.38	0.53	54	–0.57	0.57
	Post-test	30	3.46	0.55			
Self-confidence	Pre-test	30	3.30	0.61	54	–0.52	0.61
	Post-test	30	3.43	0.60			
Satisfy	Pre-test	30	3.46	0.54	54	0.39	0.70
	Post-test	30	3.37	0.60			

*p < 0.05.

#### Discussion of Differences in the Control Group Before and After Cooperative Learning

The results of this research were found to support research hypothesis 4, i.e., that heterogeneous grouping in cooperative learning can improve learning motivation. This may be due to the help of students with strong action skills, so that other group members can gain successful experience, thereby enhancing self-confidence. In addition, due to the design of the teaching plan, each student had their own goals to achieve, contributing to achievement of team goals. In order to contribute to their group, individuals had to practice harder. Cooperative learning is something that students may not have had much experience of in physical education in the past. With this new experience and fresh relationships, not just practicing alone but in a group, there are more opportunities for exchanges, encouragement and feedback between peers, which, in turn, improves learning motivation. However, the reason for post-test scores being lower than those of the pre-test, in terms of satisfaction and attention, may be the research focus on basketball, which was not the favorite sport of most students in the classes involved. In order to carry out our research, the teaching experiment had to match the progress of the teacher. A series of 12 consecutive basketball lessons is quite different from four lessons interspersed with other activities in a general teaching unit. The participants could not engage in other sports such as badminton or volleyball. Even students who liked basketball could not engage in activities such as “bullfighting,” thus depriving them of learning or engaging in other sports. The results may therefore have been affected by the limited opportunity for the type of exercise chosen as the research focus (Song et al., 2011; Chu and Chen, 2018).

#### Differences in the Experimental Group Before and After Cooperative Learning

These students were allowed greater flexibility in terms of grouping themselves. The independent sample *t*-test conducted before and after cooperative learning (see Table 10) indicated that overall learning motivation was significantly different (*t* = −2.12, *p* < 0.05), but attention, perceived relevance, self-confidence and satisfaction were not significant (*t* = 0.60, −0.90, 1.15, −0.49, *p* > 0.05). The pre-test scores for attention were higher than those in the post-test (*M* = 2.90 > 2.87); post-test scores for perceived relevance, self-confidence and sense of satisfaction were higher than those of the pre-test (*M* = 3.21 < 3.30, 2.89 < 3.10, 3.13 < 3.25).

**TABLE 10:** The number of repetitions *t*-test analysis before and after the “ARCS learning motivation scale” of the experimental group.

Variable	Test	Number	Mean	Standard deviation	Degree of freedom	*t*-Test	Significance
Learning motivation	Pre-test	29	2.88	0.30	53	–2.12	0.04*
	Post-test	29	3.18	0.42			
Attention	Pre-test	29	2.90	0.45	53	0.60	0.53
	Post-test	29	2.87	0.39			
perceived relevance	Pre-test	29	3.21	0.48	53	–0.90	0.36
	Post-test	29	3.30	0.49			
Self-confidence	Pre-test	29	2.89	0.60	53	–1.15	0.27
	Post-test	29	3.10	0.47			
Satisfy	Pre-test	29	3.13	0.55	53	–0.49	0.63
	Post-test	29	3.25	0.57			

*p < 0.05.

#### Discussion of the Differences Between Pre-test and Post-test Scores for the Experimental Group

The results of this research were found to support research hypothesis 3, i.e., that the free grouping method can improve affective partial conformity. Free grouping can help establish a harmonious atmosphere of cooperation, and a harmonious class atmosphere can enhance motivation for learning in physical education. Although overall learning motivation was found to significantly improve, other aspects (attention, perceived relevance, self-confidence, and satisfaction) were not significant. The researchers believe that due to the limit imposed on the number of groups and group size, one group was not entirely happy and had a level of unwillingness to engage. This group was composed of one student with a high level of skills and four others with lower skill levels. Because they did not usually get along very well, they often showed unwillingness to cooperate in the classroom. Some left their usual group of friends (for the sake of the research) to focus on another group of friends during the practice, which resulted in lower levels of attention, perceived relevance, self-confidence and satisfaction, and this meant that certain goals were not achieved; even attention level scores were reduced. Although students in the experimental sample were allowed to freely group themselves, it was inevitable that several individuals would be left out, meaning that single students ended up gathered together in one group. It was therefore impossible to ensure that all groups were truly “freely” selected, with group members fully aligned with one another. Indeed, it has been argued that students are maladaptive in grouping, which is echoed in grouping theory.

### Comparison of the Effectiveness of Action Skills Learning in the Control Group and the Experimental Group

The main purpose of this section is to test research question 3, i.e., to compare differences in action skills learning between the control group and experimental group.

#### Differences in Action Skills Learning Between the Control Group and Experimental Group

Performance of basketball action skills in the experimental group and the control group was found to be homogeneous in the first test. The independent sample *t*-test results for overall skill performance, shooting and passing are shown in Table 11. After analysis, it was found that the experimental group and the control group were of the same quality. The difference in the overall performance, shooting and passing tests did not reach significant levels (*t* = 1.00, −1.58, 1.81, *p* < 0.05). The experimental group was better than the control group in terms of overall performance and passing (*M* = 67.51 > 63.81, 36.30 > 32.32), and the control group was better than the experimental group in terms of shooting performance (*M* = 12.30 < 14.35). After analysis, it was found that the differences in dribbling scores between the two groups were not significant (*F* = 0.02, *p* < 0.05), and the experimental group was better than the control group (*M* = 18.30 > 17.71).

**TABLE 11:** Action Skill Performance post-measurement repetition number *t*-test analysis.

Variable	Test	Number	Mean	Standard deviation	Degree of freedom	*t-*Test	Significance
Overall technology	Pre-test	29	67.51	11.20	54	1.00	0.32
	Post-test	30	63.81	15.40			
Shooting	Pre-test	29	12.30	3.11	54	–1.58	0.12
	Post-test	30	14.35	6.03			
Passing	Pre-test	29	36.30	8.03	54	1.81	0.08
	Post-test	30	32.32	8.10			

### Discussion of Differences in Action Skills Learning Between the Control Group and the Experimental Group

The research results showed that there were no significant differences in action skills learning between the control group and the experimental group. This finding supports research hypothesis 3. The research results indicated that there was no significant difference between heterogeneous grouping and free grouping. In this study, we found that heterogeneous grouping and “free” grouping can both effectively improve the quality of students’ action skills in a cooperative learning situation, and there was no difference between heterogeneous grouping and free grouping. There was no significant improvement in the two groups in terms of “shooting.” The researchers believe that shooting may have been the first skill to be learnt after the groups were organized. The group is in the middle of the group. Differences in styles and values among group members will negatively affect group interaction. In this study, it was found that the skills of “passing” and “dribbling,” which were introduced during the first stage of the teaching unit, had been adequately honed by the time the post-tests were conducted, so a significant improvement was seen in performance of these skills. It is believed that learners must first have the opportunity to speak before they can gain knowledge through interaction and dialog, and then improve their action skills. In heterogeneous groups, due to differences in ability, high-ability group members tend to be the ones who are listened to most. For classmates, the chances of speaking when individuals are of a lower ability are reduced, and the effectiveness of their learning is likely to be compromised. Free grouping creates more opportunities for expression, and the interaction between group members is more equal than that of heterogeneous grouping, although there are also opportunities in free grouping. There are different levels of ability, but there are fewer people playing the role of leader, so they are willing to respect opinions, and there are opportunities for expression regardless of ability. Students who are grouped freely tend to think that team members have better tacit understanding and a high degree of cooperation. Members can feel the centripetal force when they discuss tasks together and cooperate in the division of labor.

It has been shown that S-type heterogeneous grouping can easily make high-ability students feel greater learning pressure, and it can cause additional burdens, but in free grouping, responsibility can be shared among group members. In addition, because dribbling and passing are relatively basic skills, the level of difficulty is not high. As long as students are willing to improve their abilities, the skills in question are not directly related to the grouping method. As long as the teaching content matches the needs of students being taught, teachers can support learning by assisting students, and most learners can achieve good results. The researchers in this study believe that the lack of significant differences in action skills learning between the experimental group and the control group may be due to the above-mentioned reasons. The two grouping methods were found to have an effect on action skills, and no difference in learning effectiveness was observed. Clearly, if the teacher is aware of students’ skill levels in physical education, there will be no need to spend one or two lessons conducting pre-tests, and this knowledge can then be used for the purposes of heterogeneous grouping. Free grouping only takes three to five minutes to organize. In either of these cases, grouping can be completed quickly, and the time saved can be used in physical education and practice (Zhang et al., 2012).

### Comparison and Discussion

The main purpose of this section is to test the fourth research question, mainly to compare differences in post-test motivation scores between the control group and the experimental group.

#### Post-test Differences in Learning Motivation Between the Control Group and the Experimental Group

The experimental group and the control group were homogenous in terms of the ARCS Motivation Scale. Overall learning motivation, perceived relevance and post-satisfaction test results are shown in Table 12. The results of the independent sample *t*-test are shown in Table 12. The analysis indicated that differences between the experimental group and the control group in terms of learning motivation, perceived relevance and post-satisfaction were not significant (*t* = −1.78, −0.56, −1.00, *p* < 0.05), and the control group was in learning motivation, perceived relevance, satisfaction The control group was better than the experimental group (*M* = 3.09 < 3.37, 3.30 < 3.41, 3.25 < 3.40).

**TABLE 12:** ARCS Learning Motivation Scale post-measurement repeat measurement *t-*test analysis.

Variable	Test	Number	Mean	Standard deviation	Degree of freedom	*t*-Test	Significance
Learning motivation	Pre-test	29	3.09	0.40	54	−1.78	0.08
	Post-test	30	3.37	0.54			
Self-relation	Pre-test	29	3.30	0.48	54	−0.56	0.56
	Post-test	30	3.41	0.52			
Satisfaction	Pre-test	29	3.25	0.58	54	−1.00	0.32
	Post-test	30	3.40	0.61			

Single-factor covariate analysis was conducted to test the different qualities of “attention” and “self-confidence,” and it was found that the “attention” aspect was not significant (*F* = 0.15, *p* < 0.05). The control group was better than the experimental group (*M* = 3.26 > 2.93), and “self-confidence” was not significant (*F* = 0.13, *p* < 0.05). The control group was better than the experimental group (*M* = 3.36 > 3.19). That is, after excluding the influence of the pre-test, there was no significant difference in learning motivation between the experimental group and the control group after receiving eight weeks of cooperative learning.

#### Discussion of Post-test Differences in Motivation Between the Control Group and the Experimental Group

The results of the study indicated that there was no significant difference in learning motivation between the experimental group and the control group after cooperative learning. The results were found to support the research hypothesis, i.e., that there was no significant difference between heterogeneous grouping and free grouping. Past studies have pointed out that contextual factors will directly affect learners’ motivation, and cooperative learning provides a context that can effectively enhance students’ learning motivation. In the context of cooperative learning, we found no significant difference between free grouping and action skills grouping. The researchers believe that the reason why free grouping can improve overall learning motivation may be that allowing students to form their own groups helps support peer group emotions and creation of a harmonious learning atmosphere. Group members are more likely to actively help one another when they are friends. However, the free grouping process might have made some students feel anxious or afraid of being left out when looking for group members, and this may have had a negative impact on the experimental group. In the action skills grouping, because each group had more capable students, who could give guidance and assistance, most of the group members were able to experience a feeling of success, so learning motivation also improved (mostly from the satisfaction of learning achievements and inner gains from success). Such rewards often help students to improve their self-confidence and motivation to learn. It should also be pointed out that students can gain self-confidence in action skills learning, and this is consistent with the self-confidence part of the ARCS learning motivation theory.

## Conclusion

This study used two different grouping methods in cooperative learning (free grouping and skills-based grouping) to teach basketball in physical education. The results in terms of learning effectiveness and learning motivation for the two groups of students were found to be relevant to school physical education. The following conclusions were reached:

(1) In cooperative learning, free grouping and action skills grouping can improve the effectiveness of skill acquisition in physical education.
(2) In cooperative learning, free grouping and skills-based grouping can effectively enhance learning motivation.
(3) In cooperative learning, no clear differences were found in terms of the effectiveness of action skills learning among self-selected groups and skills-based groups.
(4) In cooperative learning, no clear differences were found in terms of learning motivation among self-selected groups and skills-based groups.

The limitation of this study mainly is that the objective environment and content setting of teaching will affect the action skills learning motivation and produce different research results. Therefore, the teaching content and environmental factors should be unified in the research process.

It is recommended that future work should seek to explore existing relationships between students and peers so that a good cooperation mechanism can be established to promote better relationships between students.

## Data Availability Statement

The original contributions presented in the study are included in the article/supplementary material, further inquiries can be directed to the corresponding author/s.

## Author Contributions

CY: conceptualization, methodology, writing, and funding acquisition. RC: formal analysis, investigation, and supervision. XC: methodology, writing – review and editing, and funding acquisition. K-HL: validation and investigation. All authors contributed to the article and approved the submitted version.

## Conflict of Interest

The authors declare that the research was conducted in the absence of any commercial or financial relationships that could be construed as a potential conflict of interest.

## Publisher’s Note

All claims expressed in this article are solely those of the authors and do not necessarily represent those of their affiliated organizations, or those of the publisher, the editors and the reviewers. Any product that may be evaluated in this article, or claim that may be made by its manufacturer, is not guaranteed or endorsed by the publisher.

## Appendix

### Questionnaire

Student, hello! Thank you very much fortaking valuable time to fill in the questionnaire. Your help will play a vital role in my experiment, thanks for your participation!

**Table:**

Sequence number	Questions
1	Your Sex: ( )
	A.Male	B.Female
2	Do you like sports? ( )		
	A.Is	B.No
3	What sports do you like ? ( )			
	A.Basketball E.Ski I.Badminton	B.Football F.Fencing J.Table tennis	C.Volleyball G.Swimming K.Other ( )	D.Tennis H.Baseball
4	I prefer the teachers to set good teaching goals in physical education activities. ( ) A.Completely not in my case B.Not quite in my case C.Unsure D.Basically consistent with my situation E.Very fit with my situation			
5	The harder the sports skills, the more I like to try and solve it. ( ) A.Completely not in my case B.Not quite in my case C.Unsure D.Basically consistent with my situation E.Very fit with my situation			
6	No matter how my sports skills in physical learning, as long as I can get new experience, I am satisfied. ( ) A.Completely not in my case B.Not quite in my case C.Unsure D.Basically consistent with my situation E.Very fit with my situation			
7	I am very aware of my sports situation, and my purpose is to challenge myself. ( ) A.Completely not in my case B.Not quite in my case C.Unsure D.Basically consistent with my situation E.Very fit with my situation			
8	I will want to ask the teacher to show me more opportunities during class. ( ) A.Completely not in my case B.Not quite in my case C.Unsure D.Basically consistent with my situation E.Very fit with my situation			
9	I like the simple sports content that the teacher arranges and easy to learn. ( ) A.Completely not in my case B.Not quite in my case C.Unsure D.Basically consistent with my situation E.Very fit with my situation			
10	I am more concerned about what sports I learn, but what I get from. ( ) A.Completely not in my case B.Not quite in my case C.It is not clear D.Basically consistent with my situation E.Very fit with my situation			
11	I like to choose sports that I am sure of, not those that need me to do my best. ( ) A.Completely not in my case B.Not quite in my case C.It is not clear D.Basically consistent with my situation E.Very fit with my situation			
